# Low serum total testosterone level as a predictor of upstaging and upgrading in low-risk prostate cancer patients meeting the inclusion criteria for active surveillance

**DOI:** 10.18632/oncotarget.12906

**Published:** 2016-10-25

**Authors:** Matteo Ferro, Giuseppe Lucarelli, Dario Bruzzese, Giuseppe Di Lorenzo, Sisto Perdonà, Riccardo Autorino, Francesco Cantiello, Roberto La Rocca, Gian Maria Busetto, Amelia Cimmino, Carlo Buonerba, Michele Battaglia, Rocco Damiano, Ottavio De Cobelli, Vincenzo Mirone, Daniela Terracciano

**Affiliations:** ^1^ Department of Urology, European Institute of Oncology, Via Ripamonti, Milan, Italy; ^2^ Department of Emergency & Organ Transplantation - Urology, Andrology and Kidney Transplantation Unit, University of Bari, Bari, Italy; ^3^ Department of Public Health, University of Naples Federico II, Naples, Italy; ^4^ Department of Clinical Medicine, Medical Oncology Unit, University of Naples Federico II, Naples, Italy; ^5^ Department of Urology, Istituto Nazionale Tumori Fondazione Giovanni Pascale - IRCCS, Naples, Italy; ^6^ University Hospitals Urology Institute, Cleveland, OH, USA; ^7^ Division of Urology, Magna Graecia University, Catanzaro, Italy; ^8^ Department of Urology, University of Naples Federico II, Naples, Italy; ^9^ Department of Urology, La Sapienza University, Rome, Italy; ^10^ Institute of Genetics and Biophysics A. Buzzati Traverso, National Research Council, Naples, Italy; ^11^ University of Milan, Milan, Italy; ^12^ University of Medicine Iuliu Hatieganu, Cluj-Napoca, Romania; ^13^ Department of Translational Medical Sciences, University of Naples Federico II, Naples, Italy

**Keywords:** testosterone, prostate cancer, active surveillance, upgrading, upstaging

## Abstract

Active surveillance (AS) is currently a widely accepted treatment option for men with clinically localized prostate cancer (PCa). Several reports have highlighted the association of low serum testosterone levels with high-grade, high-stage PCa. However, the impact of serum testosterone as a predictor of progression in men with low-risk PCa has been little assessed.

In this study, we evaluated the association of circulating testosterone concentrations with a staging/grading reclassification in a cohort of low-risk PCa patients meeting the inclusion criteria for the AS protocol but opting for radical prostatectomy.

Radical prostatectomy (RP) was performed in 338 patients, eligible for AS according to the following criteria: clinical stage T2a or less, PSA<10ng/ml, two or fewer cancer cores, Gleason score (GS)=6 and PSA density<0.2 ng/mL/cc. Reclassification was defined as upstaging (stage>pT2) and upgrading (GS=7; primary Gleason pattern 4) disease. Unfavorable disease was defined as the occurrence of pathological stage>pT2 and predominant Gleason score 4. Total testosterone was measured before surgery.

Low serum testosterone levels (<300 ng/dL) were significantly associated with upgrading, upstaging, unfavorable disease and positive surgical margins. The addition of testosterone to a base model, including age, PSA, PSA density, clinical stage and positive cancer involvement in cores, showed a significant independent influence of this variable on upstaging, upgrading and unfavorable disease.

In conclusion, our results support the idea that total testosterone should be a selection criterion for inclusion of low-risk PCa patients in AS programs and suggest that testosterone level less than 300 ng/dL should be considered a discouraging factor when a close AS program is considered as treatment option

## INTRODUCTION

Active surveillance (AS) has recently emerged as an alternative treatment for patients with low-risk prostate cancer (PCa)-related mortality, whereby curative intervention can be delayed until the time that disease is re-classified or there is evidence of disease progression [1]. At present, urologists still suffer from a limited preoperative ability to reliably predict the tumor aggressiveness. Clinical stage, tumor grade (biopsy Gleason score) and prostate-specific antigen (PSA) are the established preoperative prognostic markers. However, despite these variables it is still difficult to determine which patients are candidates for AS. Literature data indicate a progression rate of about 30% in men on AS and a PCa-specific survival of < 97% at 5 years [2]. Thus, there is an urgent need of additional markers allowing us to discriminate between indolent or aggressive diseases.

Accumulating data indicate an important association between low testosterone concentrations and worrisome aspects of PCa. Multiple studies have reported the association of lower serum testosterone concentrations with high-grade PCa and a higher stage at presentation [3–6]. Furthermore, there is growing evidence supporting the theory that a drop in serum testosterone levels may modulate PCa risk and aggressiveness, since different metabolic disorders, such as obesity and metabolic syndrome (MetS), which are associated with low serum testosterone levels, have also been associated with an unfavorable PCa outcome [7, 8].

On this basis, total testosterone should be measured in patients with a localized PCa, in particularly when AS or nerve-sparing surgery is considered. In this study, we explored the impact of serum testosterone on upgrading, upstaging, unfavorable disease, positive surgical margins and predominant Gleason score 4 in a cohort of patients with very low-risk PCa who met the inclusion criteria for Prostate Cancer Research International: Active Surveillance (PRIAS) protocol, but elected to undergo radical prostatectomy (RP).

## RESULTS

A total of 338 patients with PCa were enrolled in this study. Demographic and clinical characteristics of the overall study population are summarized in Table 1.

**Table 1:** Clinical and Pathological Characteristics

Variable	n=338 Median [25^th^ - 75^th^ percentile]
Age, years	63.5 [59 ; 67]
PSA (ng/mL)	5.6 [4.29 ; 7.25]
PSA density	0.12 [0.09 ; 0.15]
Prostate Volume	49 [40.75 ; 55]
Familiarity, Yes	21 (6.2%)
PNI, Yes	20 (5.9%)
Nr of Positive Cores, 2	160 (47.3%)
Max % of core involved by tumor	20 [10 ; 30]
Positive DRE	36 (10.7%)
Testosterone (ng/dL)	451.5 [380 ; 566]
Testosterone <300ng/dL	53 (15.7%)

PNI, perineural invasion.

**Figure 1:**
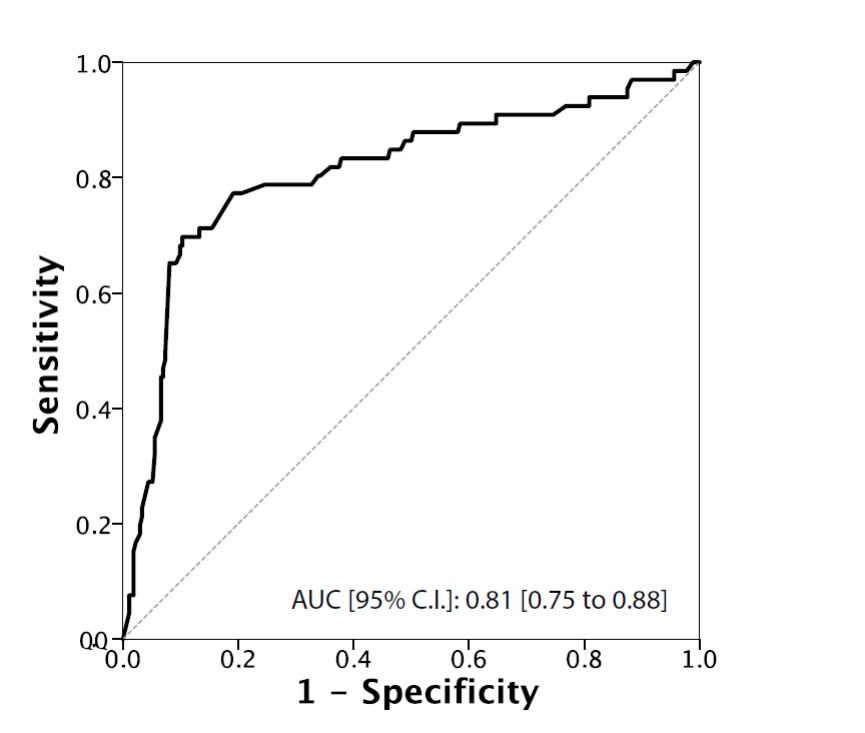
ROC Curve analysis for testosterone as a predictor of upstaging

**Figure 2:**
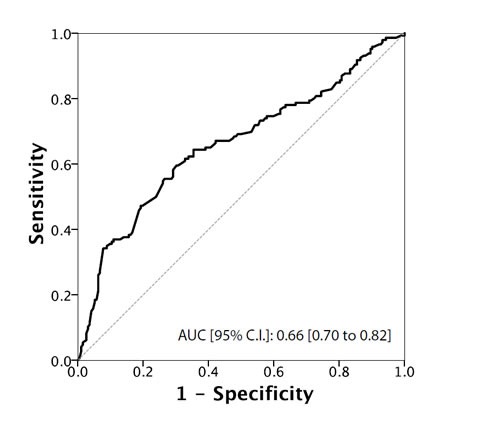
ROC Curve analysis for testosterone as a predictor of upgrading

**Figure 3:**
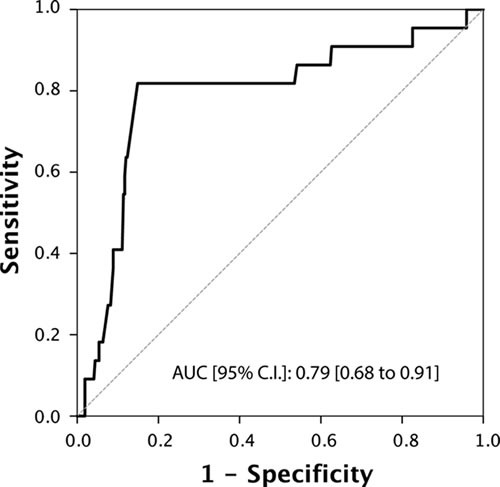
ROC Curve analysis for testosterone as a predictor of unfavorable disease

**Figure 4:**
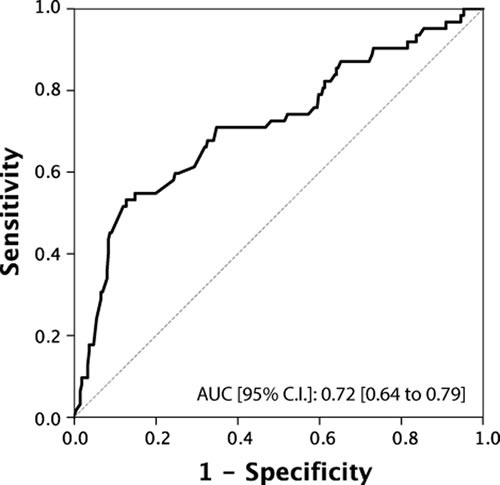
ROC Curve analysis for testosterone as a predictor of predominant Gleason score 4

Total testosterone levels showed a significant association with all the main outcomes of interest (Table 2). In particular, when treated as a continuous variable, lower total testosterone levels (median [IQR]) were associated with reclassification in terms of upstaging (299.5 [250 ; 390] *vs*. 488.5 [401 ; 600]; *p* < 0.001), upgrading (400.5 [292.25 ; 534] *vs*. 497.5 [401 ; 600]; *p* < 0.001), unfavorable disease (290 [250 ; 300] *vs*. 456 [390 ; 567]; *p* < 0.001) and predominant Gleason score 4 (300 [254 ; 502] *vs*. 477 [398 ; 597]; *p* < 0.001) . These associations were confirmed when subjects were analysed according to the presence or absence of a hypogonadism condition (total testosterone < 300 ng/dL). In addition, we found a significantly higher rate of hypogonadism in PCa patients with positive surgical margins (27.8% *vs*. 14.2%; *p* = 0.035). Of note, cancer involvement in positive cores (CIPC) was also significantly associated with all the outcomes (Table 2a and 2b). Receiver Operating Characteristic (ROC) curve analysis supported the prognostic role of total testosterone in the reclassification of men on AS (Figures 1-4). The corresponding area under the curve (AUC) ranged from 0.66 (95% C.I. 0.60 to 0.72) for upgrading, to 0.81 (95% C.I. 0.75 to 0.88) for upstaging. Sensitivities and specificities of total testosterone for each of the outcomes and according to both the “best combination” cut-off point and the cut-off point denoting a condition of hypogonadism are reported in Table 3a and 3b. To assess the role of total testosterone as an independent predictor of reclassification, a set of multivariable logistic regression models including age, PSA, PSA density, digital rectal examination (DRE) status and cancer involvement in positive cores (CIPC) was constructed (Table 4). Total testosterone included in these base models was a significant independent predictor, both as a continuous and dichotomous variable, of upstaging, upgrading and unfavorable disease. However, a significant gain in predictive accuracy was only detected for the outcome of upstaging (15.2% when considering total testosterone as a continuous variable and 12.4% when treating total testosterone as a dichotomous variable) and predominant Gleason score 4 (9.4% or 8.3%, respectively). No advantages over the base model were observed for the outcome of upgrading, unfavourable disease and for the prediction of positive surgical margins.

**Table 2A:** Clinical and pathological variables associated with tumor upstaging and upgrading. CIPC: cancer involvement in positive cores

	Upstaging			Upgrading		
	No (*n*=272)	Yes (*n*=66)	*p*-value	No (*n*=192)	Yes (*n*=146)	*p*-value
Age (years)	63.5 [59 ; 67]	63.5 [58.75 ; 66]	0.918	63 [58 ; 67]	64 [59.75 ; 67]	0.176
PSA (ng/mL)	5.68 [4.35 ; 7.3]	5.6 [4.12 ; 6.68]	0.584	5.55 [4.2 ; 7.3]	5.6 [4.43 ; 7.09]	0.834
PSA density	0.12 [0.09 ; 0.15]	0.12 [0.09 ; 0.15]	0.716	0.12 [0.09 ; 0.15]	0.12 [0.09 ; 0.15]	0.468
Prostate Volume	48 [40 ; 55]	49.5 [41 ; 54.25]	0.728	49 [41 ; 55]	48.5 [40 ; 55]	0.681
Familiarity, Yes	16 (5.9)	5 (7.6)	0.576	12 (6.3)	9 (6.2)	0.974
PNI. Yes	13 (4.8)	7 (10.6)	0.083	7 (3.6)	13 (8.9)	0.042
Nr of Positive Cores, 2	122 (44.9)	38 (57.6)	0.063	86 (44.8)	74 (50.7)	0.282
CIPC (%)	20 [10 ; 30]	40 [30 ; 50]	<0.001	20 [10 ; 30]	30 [20 ; 45]	<0.001
Positive DRE	26 (9.6)	10 (15.2)	0.186	18 (9.4)	18 (12.3)	0.477
Testosterone (ng/dL)	488.5 [401 ; 600]	299.5 [250 ; 390]	<0.001	497.5 [401 ; 600]	400.5 [292.25 ; 534]	<0.001
Testosterone <300 ng/dL	20 (7.4)	33 (50)	<0.001	13 (6.8)	40 (27.4)	<0.001

DRE: Digital rectal examination; PNI: perineural invasion; PSA: prostate-specific antigen

**Table 2B:** Clinical and pathological variables associated with unfavorable disease, positive surgical margins and predominant Gleason score 4

	Unfavorable Disease			Positive margins			Predominant Gleason 4		
	No (*n*=316)	Yes (*n*=22)	*p-*value	No (*n*=302)	Yes (*n*=36)	*p*-value	No (*n*=276)	Yes (*n*=62)	*p-*value
Age (years)	63.5 [59 ; 67]	63.5 [59.5 ; 66]	0,904	64 [59 ; 67]	61.5 [55.5 ; 65]	0.136	63 [58 ; 67]	64.5 [60 ; 68]	0,168
PSA (ng/mL)	5.6 [4.4 ; 7.3]	5.5 [4 ; 6.3]	0,244	5.64 [4.29 ; 7.25]	5.03 [4.14 ; 7.27]	0.451	5.6 [4.4 ; 7.3]	5.6 [4.1 ; 7]	0,43
PSA density	0.12 [0.09 ; 0.15]	0.11 [0.06 ; 0.14]	0,068	0.12 [0.09 ; 0.15]	0.12 [0.09 ; 0.15]	0.925	0.12 [0.09 ; 0.15]	0.11 [0.07 ; 0.15]	0,073
Prostate Volume	48 [40 ; 55]	52 [41 ; 61.3]	0,085	49 [41 ; 55]	45 [38.5 ; 53.75]	0.102	47.5 [40 ; 55]	51.5 [41 ; 60]	0,011
Familiarity, Yes	20 (6.3)	1 (4.5)	1,000	19 (6.3)	2 (5.6)	1.000	17 (6.2)	4 (6.5)	1,000
PNI, Yes	17 (5.4)	3 (13.6)	0,132	17 (5.6)	3 (8.3)	0.458	9 (3.3)	11 (17.7)	<0.001
Nr of Positive Cores. 2	146 (46.2)	14 (63.6)	0,123	146 (48.3)	14 (38.9)	0.283	129 (46.7)	31 (50)	0,642
CIPC (%)	25 [10 ; 35]	40 [30 ; 60]	<0.001	25 [10 ; 35]	30 [20 ; 40]	0.027	20 [10 ; 30]	40 [30 ; 50]	<0.001
Positive DRE	32 (10.1)	4 (18.2)	0,273	32 (10.6)	4 (11.1)	1.000	28 (10.1)	8 (12.9)	0,525
Testosterone (ng/dL)	456 [390 ; 567]	290 [250 ; 300]	<0.001	456 [389 ; 566.25]	400.5 [290 ; 540.5]	0.0643	477 [398.3 ; 597.3]	300 [254.5 ; 502]	<0.001
Testosterone <300 ng/dL	39 (12.3)	14 (63.6)	<0.001	43 (14.2)	10 (27.8)	0.035	25 (9.1)	28 (45.2)	<0.001

CIPC: cancer involvement in positive cores; DRE: Digital rectal examination; PNI: perineural invasion; PSA: prostate-specific antigen

**Table 3A:** Sensitivities and specificities of total testosterone for each of the outcomes

	Testosterone (ng/dL)	Sensitivity (95% C.I.)	Specificity (95% C.I.)
Upstaging	344	0.70 (0.59 to 0.8)	0.90 (0.86 to 0.93)
Upgrading	431	0.60 (0.52 to 0.67)	0.70 (0.63 to 0.77)
Unfavorable disease	302	0.85 (0.81 to 0.89)	0.82 (0.64 to 0.95)
Predominant Gleason 4	315	0.53 (0.4 to 0.66)	0.87 (0.83 to 0.91)

**Table 3B:** Sensitivities and specificities of testosterone < 300 ng/dL (hypogonadism) for each of the outcomes

	Hypogonadism condition		
	**Testosterone (ng/dL)**	**Sensitivity (95% C.I.)**	**Specificity (95% C.I.)**
Upstaging	300	0.65 (0.53 to 0.76)	0.92 (0.89 to 0.95)
Upgrading	300	0.34 (0.27 to 0.42)	0.92 (0.89 to 0.96)
Unfavorable disease	300	0.85 [0.81 to 0.89]	0.82 [0.64 to 0.95]
Predominant Gleason 4	300	0.52 (0.39 to 0.65)	0.88 (0.84 to 0.92)

**Table 4:** Multivariable logistic regression models including age, PSA, PSA density, DRE status and CIPC for tumor upstaging, upgrading, unfavorable disease, positive surgical margins and predominant Gleason 4

	Upstaging			
	**Continous TT**		**TT≥300ng/dL** **TT<300ng/dL**	
	**O.R. [95% C.I.]**	***p*-value**	**O.R. [95% C.I.]**	***p*-value**
Age	0.94 [0.89 to 1]	0,036	0.95 [0.9 to 1]	0,063
PSA (ng/mL)	1.22 [0.93 to 1.6]	0,149	1.15 [0.89 to 1.5]	0,29
PSA Density, 0.1 increase	0.47 [0.14 to 1.62]	0,231	0.45 [0.13 to 1.51]	0,193
Positive DRE	1.31 [0.48 to 3.55]	0,600	1.14 [0.42 to 3.12]	0,798
CIPC	1.05 [1.03 to 1.07]	<0.001	1.05 [1.03 to 1.07]	<0.001
Testosterone, 10 ng/dl increase	0.92 [0.89 to 0.94]	<0.001		
Testosterone, <300 ng/dL			11.62 [5.43 to 24.85]	<0.001
				
AUC, [95% C.I.]	0.84 [0.79 to 0.9]		0.81 [0.75 to 0.88]	
Gain in predictive accuracy; % (p-value)	15.2 (<0.001)		12.4 (0.002)	
	**Upgrading**			
	**Continous TT**		**TT≥300ng/dL** **TT<300ng/dL**	
	**O.R. [95% C.I.]**	***p*-value**	**O.R. [95% C.I.]**	***p*-value**
Age	1.01 [0.96 to 1.05]	0,827	1.01 [0.96 to 1.05]	0,791
PSA (ng/mL)	1.02 [0.84 to 1.24]	0,868	1.02 [0.83 to 1.24]	0,87
PSA Density, 0.1 increase	1.07 [0.43 to 2.63]	0,890	0.97 [0.39 to 2.39]	0,945
Positive DRE	1.18 [0.54 to 2.6]	0,674	1.14 [0.51 to 2.53]	0,754
CIPC	1.05 [1.03 to 1.07]	<0.001	1.05 [1.03 to 1.07]	<0.001
Testosterone, 10 ng/dl increase	0.97 [0.96 to 0.99]	0,001		
Testosterone, <300 ng/dL			3.6 [1.74 to 7.46]	0,001
				
AUC, [95% C.I.]	0.73 [0.68 to 0.79]		0.73 [0.68 to 0.79]	
Gain in predictive accuracy; % (p-value)	0.1 (0.935)		0.1 (0.922)	
	**Unfavorable Disease**			
	**Continous TT**		**TT≥300ng/dL** **TT<300ng/dL**	
	**O.R. [95% C.I.]**	***p*-value**	**O.R. [95% C.I.]**	***p*-value**
Age	0.95 [0.88 to 1.03]	0,217	0.95 [0.87 to 1.03]	0,197
PSA (ng/mL)	1.29 [0.86 to 1.93]	0,222	1.26 [0.82 to 1.92]	0,287
PSA Density, 0.1 increase	0.14 [0.02 to 0.87]	0,035	0.12 [0.02 to 0.88]	0,037
Positive DRE	1.23 [0.34 to 4.47]	0,754	1.03 [0.27 to 3.94]	0,968
CIPC	1.05 [1.02 to 1.08]	0,001	1.05 [1.02 to 1.08]	0,002
Testosterone, 10 ng/dl increase	0.93 [0.89 to 0.97]	0,001		
Testosterone, <300 ng/dL			10.26 [3.54 to 29.76]	<0.001
				
AUC, [95% C.I.]	0.84 [0.77 to 0.92]		0.84 [0.73 to 0.95]	
Gain in predictive accuracy; % (p-value)	6.3 (0.119)		5.9 (0.101)	
	**Positive margins**			
	**Continous TT**		**TT≥300ng/dL** **TT<300ng/dL**	
	**O.R. [95% C.I.]**	***p*-value**	**O.R. [95% C.I.]**	***p*-value**
Age	0.95 [0.89 to 1.01]	0,075	0.95 [0.89 to 1.01]	0,072
PSA (ng/mL)	0.85 [0.63 to 1.15]	0,295	0.85 [0.63 to 1.14]	0,269
PSA Density, 0.1 increase	1.85 [0.5 to 6.83]	0,358	1.79 [0.49 to 6.53]	0,379
Positive DRE	0.85 [0.26 to 2.7]	0,776	0.84 [0.27 to 2.69]	0,774
CIPC	1.02 [0.99 to 1.04]	0,191	1.02 [0.99 to 1.04]	0,205
Testosterone, 10 ng/dl increase	0.98 [0.96 to 1]	0,097		
Testosterone, <300 ng/dL			2.25 [0.94 to 5.43]	0,07
AUC, [95% C.I.]	0.66 [0.56 to 0.76]		0.66 [0.57 to 0.75]	
Gain in predictive accuracy; % (p-value)	0.74 (0.934)		1.11 (0.904)	
	**Predominant Gleason 4**			
	**Continous TT**		**TT≥300ng/dL** **TT<300ng/dL**	
	**O.R. [95% C.I.]**	***p*-value**	**O.R. [95% C.I.]**	***p*-value**
Age	1 [0.95 to 1.06]	0,996	1 [0.94 to 1.06]	0,916
PSA (ng/mL)	1.3 [0.99 to 1.71]	0,054	1.3 [0.99 to 1.71]	0,058
PSA Density, 0.1 increase	0.16 [0.05 to 0.56]	0,004	0.13 [0.04 to 0.49]	0,002
Positive DRE	0.86 [0.32 to 2.28]	0,755	0.72 [0.26 to 2.05]	0,543
CIPC	1.06 [1.04 to 1.08]	<0.001	1.06 [1.04 to 1.08]	<0.001
Testosterone, 10 ng/dl increase	0.96 [0.93 to 0.98]	<0.001		
Testosterone, <300 ng/dL			6.66 [3.14 to 14.15]	<0.001
AUC, [95% C.I.]	0.81 [0.75 to 0.87]		0.82 [0.75 to 0.89]	
Gain in predictive accuracy; % (p-value)	8.3 (0.046)		9.4 (0.041)	

For sake of readability of the results, when treating Testosterone as continuous predictor, the Odds Ratio have been computed for a 10ng/dl increase in TT levels. Testosterone has been analyzed both as continuous and as dichotomous predictor using 300ng/dl as cut-off. CIPC: cancer involvement in positive cores; DRE: Digital rectal examination; PNI: perineural invasion; PSA: prostate-specific antigen; TT: total testosterone

## DISCUSSION

The principal aim of an AS program is to reduce over-treatment in patients with clinically confined, very-low-risk PCa, without compromising curative treatment [9]. The identification of these low risk PCa is still a critical issue today, and many markers have been identified for selecting candidates for non-aggressive therapies [10–16]. Although the selection criteria for AS include stringent clinicopathological parameters, it is well established that about a third of men on AS will undergo progression requiring active treatment [1, 17]. Therefore, there is a strong interest in finding risk factors for reclassification and progression, particularly in men with a long life expectancy [18]. Conflicting results have been reported on the risk/benefit ratio of AS as upfront treatment strategy allowing radical treatment to be delayed or avoided [19–22]. Therefore, an important focus for AS protocols is to improve the selection of patients at the time of inclusion in order to minimize the reclassification of risk during follow-up. In this context, several tools have been proposed to overcome these limitations of current AS protocols. Multiparametric magnetic resonance imaging (MRI) [23], urinary prostate cancer antigen 3 (PCA3) and serum markers [24], histopathological [25–27] and genetic factors [28–30] have been analyzed.

Little is yet known about the clinical utility of serum testosterone levels as a predictor of disease reclassification in men on AS. Recently, in a relatively small population San Francisco et al [31] showed that free, but not total testosterone levels nor the free testosterone/total testosterone ratio or the testosterone/PSA ratio, were significantly lower in men with PCa and disease reclassification during AS. A number of previous reports identified a significant relationship between a high Gleason score and low testosterone levels [32–35]. Furthermore, growing evidence supports the idea that a decreased serum testosterone concentration, related to different metabolic disorders including obesity and metabolic syndrome, may modulate PCa aggressiveness [36].

We assessed the use of serum total testosterone as a predictor of disease reclassification in a cohort of men eligible for AS according to PRIAS criteria. Our results showed that men who underwent reclassification had significantly lower serum total testosterone levels compared to those who were not reclassified (*p* < 0.001). By ROC curve analysis, we identified a testosterone threshold of 344 ng/dL, 431 ng/dL, 302 ng/dL and 315 for upstaging, upgrading, unfavorable disease and predominant Gleason score 4 respectively. Men with testosterone levels lower than these values had a higher risk of disease reclassification. When the threshold value for hypogonadism ( < 300ng/dL) was used as cut-off [6, 37], we found significantly more patients with upstaging, upgrading, unfavorable disease, positive surgical margins and predominant Gleason score 4 compared to eugonadal patients. Multivariate analysis indicated that serum total testosterone, used either as a continuous or a dichotomous variable, was an independent predictor of upgrading, upstaging, unfavorable disease and predominant Gleason score 4.

Pichon et al [33] had shown in a larger study population that a low serum testosterone level was an independent predictor of a predominant Gleason pattern 4 at radical prostatectomy and of upgrading from low- to high-grade PCa between needle biopsies and prostatectomy specimens. Accordingly, the San Francisco et al study [31] showed that men on AS with low serum free testosterone levels had more than four times the risk of disease reclassification, suggesting the need for further studies to assess testosterone as a tool to better select patients for AS.

Collectively, our results suggest the importance of taking into account hypogonadism and metabolic disorders such as obesity and metabolic syndrome, as selection criteria for patients inclusion in AS programs. Consistently, the majority of large observational series have shown that obesity is a risk factor for adverse pathologic features, a more advanced stage, higher risk for biochemical recurrence after RP, and risk of death from PCa [7, 38, 39].

Our findings support the use of total testosterone as a predictor of disease reclassification for men with PCa undergoing AS. Moreover, our models including testosterone showed a significant gain in predictive accuracy of upstaging and unfavorable disease.

These data are consistent with the idea that patients with low serum testosterone levels are more likely to have aggressive PCa. AS protocols in these patients should ensure close monitoring of PSA levels and imaging examinations, in order to identify tumor progression as early as possible.

The strength of this study is the large population (338 patients on AS), allowing a robust statistical analysis. Furthermore, as recommended by the Endocrine Society guidelines, all blood samples for testosterone were analyzed using the same platform, and were collected between 07:00 AM to 10:00 AM hours, in order to avoid circadian variations and inter-assay variability. Total testosterone was measured with one of the best electrochemiluminescent immunoassays and the histological analysis of prostate biopsies and prostatectomy specimens was done by three senior uropathologists avoiding variability in Gleason scores interpretation. Moreover, exclusion criteria were strictly defined to rule out patients that had received neoadjuvant hormonal therapy or affected by other comorbidities that can affect the testosterone levels.

However, some limitations have to be taken into account. Firstly, our study was carried out retrospectively, and several useful measures that affect testosterone values, such as Sex Hormone Binding Globulin (SHBG), luteinizing hormone and oestradiol, were not determined. In addition, like previous reports on the association between hypogonadism and a poor outcome in PCa, we did not use mass-spectrometry-based measurements, recently advocated as the gold standard for sex steroid quantifications [40]. Finally, we lacked data about free and bioavailable testosterone, which were demonstrated to be important in a previous report [31]. The focus was primarily on the pathological findings, but we did not assess biochemical recurrence or prostate cancer-specific mortality, which might be a more important issue than the adverse pathological characteristics to better define progression. In fact, it is well accepted that disease reclassification may be the result of sampling error [41].

In conclusion, men with hypogonadism eligible for AS are at higher risk of disease upgrading and upstaging compared to men with normal serum testosterone levels. These results highlight the utility of evaluating testosterone levels in patients with localized PCa, eligible for AS. Further prospective studies on large populations, with mass-spectrometry-based testosterone measurements and SHBG, luteinizing hormone, oestradiol, free and bioavailable testosterone data are needed to confirm our findings and support the use of circulating sex hormones as prognostic biomarkers in patients eligible for AS.

## MATERIALS AND METHODS

### Patients

Between January 2009 and December 2015, 338 consecutive men were referred for localized PCa, and underwent, within 3 months of diagnosis, laparoscopic or robot-assisted laparoscopic RP at three tertiary care institutions (Departments of Urology of the National Cancer Institute “Fondazione Pascale”-Naples, of the University of Catanzaro and of the University of Bari).

Patients fulfilled the inclusion criteria for the PRIAS protocol [42] defined as: clinical stage T2a or less, PSA < 10 ng/ml, 2 or less cancer involvement cores after a biopsy scheme of at least 12 cores, Gleason score (GS)≤6 and PSA density < 0.2 ng/mL/cc .

Pathological findings in prostate biopsies were compared with pathological specimens after RP.

Reclassification was defined as disease upstaging (pathological stage>pT2) and upgrading (GS ≥ 7; primary Gleason pattern 4). Unfavorable disease was defined as the occurrence of pathological stage>pT2 and predominant Gleason score 4.

RP specimens were processed and evaluated according to the Stanford protocol [43] by three experienced genitourinary pathologists, blinded to the index-tests results of each Institution.

For all patients, at least 12 core biopsies were analyzed according to the 2005 International Society of Urological Pathology recommendations [44]. None of the study patients received neoadjuvant hormonal therapy (antiandrogens or luteinizing hormone-releasing hormone analogues or antagonists) or other hormonal preparations (i.e., 5-α reductase inhibitors) that could alter their PSA values. We also excluded patients with acute bacterial prostatitis or previous prostate surgery in the 3 months before biopsy. In addition, subjects with chronic renal disease, marked alterations in blood protein levels, hemophilia, incurable endocrine diseases or those who had previously undergone multiple transfusions, were excluded from the study because these conditions could alter the concentration of total PSA and testosterone.

Data collected included age, preoperative PSA level, PSA density, clinical stage and preoperative serum testosterone levels.

The threshold for hypogonadism was set at a total testosterone level of 300 ng/dL, in agreement with the American Association of Clinical Endocrinologist guidelines [37]. Accordingly, patients were further divided into two groups: 1) low total testosterone group ( < 300 ng/dL) and 2) normal testosterone group (≥300 ng/dL). Clinical stage was assessed by digital rectal examination and magnetic resonance imaging by the attending surgeon according to TNM staging (2009). Disease upstaging was regarded as pathological stage ≥T3a after RP with clinical stage ≤T2c. Prostate cancer upgrading was defined as GS ≥7 in RP specimens with GS ≤6 in needle biopsies.

This study received approval from the local hospital ethics committee (i.e. institutional review board approval). Written informed consent was obtained from all patients.

### Hormonal assay

All patients underwent systematic blood sampling between 7 AM and 10 AM on the day before surgery to assess serum total testosterone concentrations.

Electrochemiluminescence immuno-assays, using high-affinity monoclonal antibodies, were performed at the laboratories of the three Institutions, blinded to the pathological results, using Testosterone ElecsysII (Modular Analytics E170 -Roche, Basel, Switzerland).

### Statistical analysis

Numerical variables were recorded and analysed as median [25^th^ - 75^th^ percentile] while categorical variables were expressed as frequencies and percentages. Comparisons between groups were based on the Mann-Whitney or Chi square tests. The predictive accuracy of testosterone was evaluated using Receiver Operating Characteristic (ROC) analysis and quantified in terms of Area Under the Curve (AUC) and corresponding 95% confidence interval (95% C.I.). The independent role of testosterone in predicting pathological outcomes at radical prostatectomy was assessed using multivariable logistic regression models; a bootstrap approach, based on 1999 bootstrap replications, was used to compare the percentage change in predictive accuracy (in terms of AUC) between nested logistic models.

Statistical analyses and modelling were performed with R statistical computing software (R Foundation for Statistical Computing, Vienna, Austria). *P*-values < 0.05 were considered statistically significant.
